# On the Diagnosis of Phthisis Pulmonalis; Being an Extract from a Lecture Delivered at the General Dispensary, Louisville, Ky.

**Published:** 1844-08

**Authors:** J. R. Buck

**Affiliations:** Lecturer on Diseases of the Chest, in the Louisville Summer School of Medicine; Louisville


### WESTERN JOURNAL

OF

### MEDICINE AND SURGERY.

AUGUST, 1844.

### Art. I.—*On the Diagnosis of Phthisis Pulmonalis;* being an extract from a Lecture delivered at the General Dispensary, Louisville, Ky. By J. R. Buck, M.D., Lecturer on Diseases of the Chest, in the Louisville Summer School of Medicine.

In the man, Quiligan, examined in your presence at the last lecture, and now under treatment, phthisis pulmonalis is presented in what is termed by authors its first stage; in the female, Mrs. W., and in Mason, the disease is in its third or last stage.

To a methodical investigation of this suoject it is essential, that we should first settle in our minds what constitutes the disease: what is phthisis? Is it that peculiar condition of the lungs in which there is deposited in them tuberculous matter?

or is it that condition of the *system* predisposing to the deposition of this matter? The answer to this question is of the greatest practical importance; it is the pivot upon which our views of the pathology as well as the treatment of the disease must turn. If we regard tubercles in the lungs as the cause and characteristic of phthisis, as it is regarded by Louis and many other distinguished pathologists, then we have only to ascertain the presence or absence of this matter in the lungs, to determine the absence or presence of the disease. But if, on the contrary, we regard this deposit as but an effect or sign of the disease, as an index pointing us to a constitutional disease, then we are led to inquire whether there be any diagnostic measures by which the disease may be detected previous to the deposition of this matter; for whatever we may think of the possibility of curing consumption when there exists tubercles in the lungs, we must all admit that under the most favorable circumstances and the most judicious treatment, it is a most formidable disease, and too often baffles the skill of the physician.

Adopting this latter opinion then, that this deposit is but an effect of a constitutional disease, and that this disease may be detected previous to the deposit, when the disease is entirely under the control of therapeutic and hygienic measures, I proceed to the enumeration of those facts and circumstances which indicate its existence in this stage.

In treatises upon this subject you will find mentioned as predisposing causes of phthisis, the existence of the disease in the ancestors of the patient; narrowness of the chest, transparency of the skin, sedentary habits, occupations exposing to the inhalations of dust or vapor, certain ages, morbid sensitiveness of the mucous membranes; hæmoptysis. These circumstances, except hemoptysis, are generally regarded as merely indicating a predisposition to phthisis; while according to the view I entertain they indicate to us, unerringly, the actual existence of the disease, and its existence in that stage in which it is most important for us to recognise it, as being the only one in which we can hope, uniformly to effect a cure.

I am aware that Louis regards hæmoptysis, when unaccompanied by suppression of catamenia, or not the result of an injury, as indicating always the existence of tubercles in the lungs; and this he infers from the fact, that in the examination of some hundreds of subjects where hæmoptysis had occurred during some period in their lives, tubercles were found in their lungs. But is this a logical conclusion? At best it is but a remarkable coincidence; and if the uniformity of concomitance be such as to induce a belief that they sustain to each other the relation of cause and effect, where is the shadow of reason for regarding tubercles rather than hæmoptysis as the cause. Hæmoptysis occurs generally as the very first evidence of a departure from health, and long before there is the slightest trace of tuberculous deposit to be detected in the lungs by the nicest physical examination. From the limited space in which almost uniformly it is at first deposited, the thin lamina of lung constituting its summit, a very minute portion gives rise to dullness on percussion, and to certain alterations in the respiratory sounds.

If tubercles were the cause of this hemorrhage, as the deposit increased we should expect the hemorrhage to increase in the same ratio. The very reverse of this is true; it is most copious when there is no physical alteration of the lung at all; and it diminishes as the deposit increases. It is true that we sometimes have copious hæmoptysis in the last stage of phthisis. It is then caused, however, by the tubercular matter producing inflammation and ulceration of the coats of the blood-vessels—causes which cannot exist in the earlier stages. There are but two ways besides this by which tubercles can possibly produce hæmoptysis. They may by irritation cause an afflux of blood to that part of the lung; or they may, by mechanically obstructing the circulation, cause congestion, and hæmoptysis may be the result of this congestion; and doubtless this does sometimes occur, for we find slight hæmoptysis occurring occasionally throughout the whole course of tuberculous disease of the lungs.

But whenever hemorrhage is an effort of nature to relieve a congested organ, the very moment the organ is relieved the hemorrhage ceases. Can this be the case in phthisis pulmonalis? The summit of either lung cannot contain an additional half ounce of blood, without being plainly and readily detected by physical examination; and yet hæmoptysis occurs when there is no physical alteration appreciable, and then often to the extent of 5, 10, 15, or 20 ounces. But you may ask me, if this be not the cause, what is? and I candidly confess my inability to answer this question, even to my own satisfaction. It is doubtless some lesion of the blood itself, and not of the blood-vessels; in what, precisely this alteration consists has yet to be determined. It is sufficient, however, for my present purpose, to prove to you that tubercular matter is not the cause. I am endeavoring now to diagnosticate to you the existence of phthisis pulmonalis previous to the deposition of tubercles in the lungs; and hæmoptysis I regard as by far the most important sign of the disease in this stage; and as it occurs often as the very first evidence of a departure from health, it becomes exceedingly valuable as affording us the earliest information of the most insidious approach of this arch enemy of life. It may also be asked, why hæmorrhage occurs from the lungs rather than from any other organ of the body, if it result from a lesion of the blood. I do not know that any satisfactory explanation of this fact has been given. Some very recent discoveries of Professor Horner may throw some light upon this hitherto obscure subject, if not indeed free it from all obscurity. By a series of interesting experiments he has ascertained that there is direct communication between the bronchial tubes and the blood-vessels of the lungs; though the fact was at first discovered accidentally the experiments were afterwards repeated, and uniformly with the same result. While the blood is unchanged, retaining its natural cohesiveness, the circulation goes on normally, but if it lose this characteristic and becomes thin, it passes from the veins and enters into the bronchial tubes, and is thence discharged from the lungs. If a patient come to you then, from twenty to thirty years of age, the period at which the disease is most likely to occur, of sedentary habits, narrow chest, transparent skin, morbid sensitiveness to cold; whose occupation exposes him to the inhalations of dust or irritating vapor, and who has had an attack of hæmoptysis, you may pronounce it unhesitatingly a case of phthisis pulmonalis; and if you now suffer your patient to remain without treatment, tubercles will sooner or later be deposited, and the chance for a permanent cure be very materially lessened. Nor is it necessary that in each individual case all the symptoms here enumerated should be present. Take any other disease, pneumonia, pleurisy, or pericarditis, and you meet with cases where one or two or more of what are termed the most prominent symptoms are absent; so it is with the disease under consideration, although in every case a sufficient number of these symptoms remain to characterize the disease. At this stage of phthisis the treatment is simple and such as would naturally suggest itself to every intelligent practitioner. A change in the habits and mode of living of the patient will often be sufficient to effect a cure. If medicines are required at all, gentle tonics, of which the ferruginous preparations are the best, with purgatives, diuretics and diaphoretics, as occasion may require, will be found amply sufficient.

I have here notes of three cases, taken from my case book, in which the symptoms of phthisis presented themselves years before there was the slightest evidence of disease of the lungs; and when tuberculous deposition might, in all probability, have been prevented.

Miss T. æt. 18 years, dark hair and eyes, habits sedentary, chest narrow. Father, brother, and sister died of phthisis pulmonalis. Miss T. enjoyed uninterrupted health until 15 years of age, when, without any apparent disease, she became slightly debilitated, not sufficiently so, however, to be confined to the house. While in this condition she exerted herself in attempting to prevent an acquaintance from getting a letter out of her hand, and was a few minutes afterwards seized with hæmoptysis, amounting in the three days during which it continued to several pints. Alum, acetate of lead, and acid drinks were administered. In the course of a few weeks she was restored to her usual health, and with the exception of repeated colds was not again in the slightest manner indisposed till June, 1842—just two years and a half subsequent to the hæmoptysis. An attack of cold then left her with a short dry cough, and occasionally some slight shooting pains about the chest. These symptoms continued for several months, when she was attacked with chills, first every other day, and then every day, followed by fever and profuse night sweats, and total suppression of catamenia.

I first saw her October 25th, 1842. She then had cavities in the summit of the left lung and tubercular matter in the summit of the right. She died January, 1843.

Andrew Spencer, æt. 30 years, a cooper by trade, tall, very spare, coal-black hair, black eyes, no family predisposition to disease of any kind; had intermittent fever occasionally when a boy; with that exception his health had always been good. July, 1839, while at work at his trade, apparently in perfect health, hæmoptysis to the extent of a pint came on; this continued, at intervals of a few weeks, sometimes of months, for two years, without the slightest evidence of disease of the lungs or of any other organ of the body. Symptoms of a local affection of the lungs then appeared. When I first saw him, July 13th, 1842, cavities existed in both lungs.

Mrs. B., æt. 43 years, below medium height, dark complexion, very slight family predisposition to phthisis pulmonalis, health always good until she was 39 years of age, when she had an attack of pleurisy. She recovered from the pleurisy perfectly, but has always since been remarkably susceptible to cold—to use her own language, she "hardly knew what it was to be without a cold." Three years after the pleurisy, while in the enjoyment of her usual health, and, what alarmed her still more, while free from cold, she suddenly felt a tickling sensation in the throat, giving rise to slight cough, with the expectoration of about half a pint of blood. From this she recovered in a few days, and continued free from any symptom of disease of the lungs for two years. In January, 1844, it was discovered that there were cavities in the left lung, and tubercular matter in the right. She died in March.

I might mention other similar cases, many of which doubtless have occurred in the practice of almost every physician, as they will also occur in yours.

It is much to be regretted that while we possess the means of detecting the disease at this stage, we are rarely called upon to do so. If the physician is called in at all, it is only to treat an attack of hæmoptysis; and so soon as that is relieved his services are dispensed with. If he should represent to the patient his true condition and the threatening nature of the disease, his opinion, generally, would be disregarded. Patients will not disconnect consumption from cough expectoration, pain in the breast, &c., and so long as they have no symptoms of a local affection of the lungs, so long will you be unable to succeed in convincing them, that they have consumption. Hence it becomes important that the distinction between tubercles in the lung, and phthisis be known and recognized by all; that patients may be induced to submit to treatment when their diseases may be readily cured. It is also essential to a judicious treatment, that this distinction be borne in mind—that the disease be separated from one of its effects, more especially as the effect is opposite in character to the disease. The disease being constitutional debility, the effect local inflammation, remedies for one condition may act injuriously upon the other, if injudiciously administered.

Louisville, July, 1844.

